# The impact of nursing intervention on the quality of life of patients with major depressive disorder: a narrative review

**DOI:** 10.3389/fpsyt.2025.1689832

**Published:** 2025-11-19

**Authors:** Zhizhen Liao, Wene Liu, Luojuan Yin

**Affiliations:** The First Hospital of Hunan University of Chinese Medicine, Hunan, China

**Keywords:** nursing intervention, quality of life, major depressive disorder, psychoeducation, cognitive-behavioral therapy

## Abstract

Major Depressive Disorder (MDD) profoundly impairs patients’ quality of life (QoL), creating a critical gap between symptom remission and holistic functional recovery. This narrative review examines the potential role and conceptual basis of nursing interventions in addressing this gap and improving QoL for individuals with MDD. It explores the evolution of mental health nursing from custodial care to a recovery-oriented, therapeutic practice, emphasizing the nurse’s unique position in providing continuity within fragmented care systems. The review classifies and discusses key nursing strategies—including psychoeducation, cognitive-behavioral techniques, integrated case management, mind-body interventions, and social reintegration support—while critically appraising the strength of the available evidence. A central theme throughout is the methodological challenge that much of the existing literature infers QoL benefits from symptom or functional improvement, rather than consistently employing validated, multidimensional QoL measures as primary outcomes. The review also elucidates proposed mechanistic pathways, such as the therapeutic alliance and self-efficacy development, through which nursing care may enhance QoL. Significant barriers to implementation, including workforce shortages and role ambiguity, are addressed. We conclude that while nursing interventions hold significant potential to improve patient-reported QoL, this potential is not yet fully realized or evidenced. Future work must prioritize the systematic embedding of standardized QoL assessment into intervention research, validate technology-driven solutions, and conduct robust trials to firmly establish the impact of nursing care on the holistic well-being of individuals living with MDD.

## 1 Introduction

Major Depressive Disorder (MDD) is a pervasive mental health condition affecting over 280 million people globally, with significant implications for healthcare systems and societal burden (1). MDD profoundly impairs patients’ quality of life (QoL), extending beyond symptom reduction to encompass functional, social, and emotional well-being (2). While pharmacological and psychotherapeutic treatments target symptom remission, the disconnect between clinical recovery and holistic QoL improvement remains a critical challenge (3). Nursing interventions, emphasizing continuity and patient-centered care, potentially bridge this gap (4). However, existing reviews often focus narrowly on specific interventions or outcomes, lacking comprehensive synthesis of how diverse nursing strategies collectively impact QoL in MDD. Despite growing recognition of nursing’s role in mental health, a comprehensive synthesis focusing specifically on how nurse-led interventions influence quality of life in MDD remains limited. This review aims to synthesize evidence on nursing interventions’ impact on QoL in MDD, analyze mechanistic pathways, identify implementation barriers, and propose future directions for research and practice.

Nursing practices, evolved from custodial care to proactive therapeutic models, are uniquely positioned to address these dimensions through psychoeducation, cognitive-behavioral techniques (CBT), and integrated care pathways (5, 6). Nurse-led case management has shown promise in improving treatment adherence and reducing stigma-related isolation, directly influencing QoL outcomes (7, 8). Studies within mental health settings have demonstrated that nurse-delivered psychosocial interventions, including those focusing on structured activity and family support, can enhance emotional regulation and facilitate occupational reengagement, which are key determinants of QoL in MDD (9, 10). However, barriers such as workforce shortages and inconsistent outcome measurement hinder implementation (11). By evaluating mechanistic pathways—such as therapeutic alliance and self-efficacy development—this article underscores nursing’s role in fostering empowerment and resilience (12, 13).

Innovations like digital platforms and standardized nursing protocols offer scalable solutions, yet their integration into MDD care pathways requires further evidence (8). This narrative review highlights the need for interdisciplinary collaboration to optimize QoL-centric interventions. Given the current state of evidence, which often infers QoL improvements through proxy measures rather than consistently employing validated QoL instruments, this article aims to synthesize the conceptual arguments and preliminary evidence that support the potential of nursing care to enhance QoL. Ultimately, we advocate for nursing practices to be systematically embedded in MDD care and for future research to more directly and rigorously evaluate their impact on patient-reported QoL.

## 2 Methods: literature search strategy for this narrative review

This article is a narrative review. Its primary aim is to provide a comprehensive and critical synthesis of the existing literature on the role of nursing interventions in enhancing the QoL of patients with MDD. The objective is to explore conceptual frameworks, classify interventions, elucidate mechanistic pathways, and discuss implementation challenges, rather than to conduct a systematic aggregation of all available evidence.

The literature search and selection for this review were conducted in a non-systematic manner, focusing on breadth and conceptual relevance to build a coherent narrative. To ensure comprehensive coverage of the topic, the following strategy was employed:

### 2.1 Electronic databases and search terms

Searches were conducted in the electronic databases PubMed, CINAHL, and PsycINFO for relevant English-language publications. Combinations of key terms were used, including: (“Major Depressive Disorder” OR “depression”) AND (“nursing intervention” OR “nurse-led” OR “psychiatric nursing”) AND (“quality of life” OR “QoL” OR “well-being”).

### 2.2 Time frame and selection criteria

The search was not restricted by a rigid time to capture both foundational theories and recent advancements. Articles were selected based on their relevance to the core themes of the review, including conceptual models of QoL in MDD, the historical evolution of mental health nursing, specific nursing interventions (psychoeducation, cognitive-behavioral therapy [CBT], case management), and barriers to care. We prioritized peer-reviewed original research, review articles, and clinical guidelines. The selection process was iterative and involved examining the reference lists of key articles (snowballing method) to identify additional relevant publications.

It is important to note that, as a narrative review, this work does not claim to be exhaustive. The synthesis and interpretation of the literature are inherently influenced by the authors’ perspective and the goal of building a thematic narrative. Therefore, some selection bias may be present. The findings and conclusions should be interpreted as a critical analysis and discussion of the existing landscape, intended to highlight key insights, gaps in knowledge, and directions for future research.

#### 2.2.1 Conceptualizing QoL in MDD: beyond symptomatology

MDD is a complex mental health disorder that extends beyond the presence of clinical symptoms such as low mood, anhedonia, and fatigue. QoL in MDD encompasses a broader spectrum of psychological, physical, social, and functional dimensions. While symptom reduction remains a primary treatment goal, it does not always correlate with improved QoL, necessitating a more holistic approach to patient care (14). The WHO defines QoL as an individual’s perception of their position in life within the context of their culture, value systems, and personal goals. This definition underscores the subjective nature of QoL, which may differ significantly among individuals with similar symptom severity (15–17). A patient with mild depressive symptoms may report severe QoL impairment due to occupational dysfunction, whereas another with severe symptoms may adapt better socially, highlighting the need for personalized assessments (18, 19).

##### 2.2.1.1 Multidimensional constructs of QoL in chronic mental illness

MDD often leads to persistent QoL deficits that persist beyond acute symptomatic episodes. The WHOQOL-Bref instrument captures four key domains—physical health, psychological well-being, social relationships, and environment—providing a comprehensive framework for evaluating QoL in MDD (17, 20). Evidence shows that individuals with MDD often place greater value on psychological and social aspects than on physical health when rating their QoL (18, 21). Depressive states tend to shift the interpretation of WHOQOL-Bref items toward emotional rather than physical priorities (18). The primacy of psychosocial factors in determining QoL is also observed in other chronic conditions with high rates of comorbid depression. For instance, in end-stage renal disease, the strength of social support can be a more powerful predictor of QoL than some biological markers (22, 23). This reinforces the broader principle that in chronic illness states—including chronic mental illness like MDD—psychosocial variables often dominate the subjective lived experience of well-being.

##### 2.2.1.2 The discrepancy between symptom remission and functional recovery

Symptom remission, as measured by instruments such as the Hamilton Depression Rating Scale (HAMD), does not inherently equate to a return to full functional capacity. Between 30% and 50% of individuals who reach remission continue to face limitations in daily activities, work performance, and social interaction (24, 25). In outpatient MDD populations, somatic complaints—including chronic pain—correlate more strongly with QoL impairment than mood disturbances alone (25). This aligns with findings from stroke rehabilitation, where post-stroke MDD was the primary determinant of QoL decline, surpassing physical disability (26). Longitudinal observations confirm that improvement in QoL early in the course of treatment is a predictor of eventual symptom remission, irrespective of initial severity (19). This suggests that interventions targeting functional and psychosocial outcomes may enhance long-term recovery, even in patients with residual symptoms (27, 28).

##### 2.2.1.2 Relevance of the WHOQOL model in evaluating holistic outcomes in MDD

The WHOQOL-Bref’s bifactor structure—validated across diverse populations, including psychiatric patients—makes it particularly suited for MDD care (17, 20). Its emphasis on subjective perception aligns with recovery-oriented models, which prioritize patient-defined goals over clinician-rated metrics (21, 28). Critically, the tool’s mental health recovery module (WHOQOL-MHR) addresses gaps in assessing hope, empowerment, and autonomy—key themes in MDD recovery (21). Achieving QoL parity with the general population in primary care MDD settings appears possible only when both depressive and anxiety symptoms are addressed, reinforcing the need for a multidimensional and integrated approach to assessment and treatment (29). Circadian rhythm disturbances, commonly observed in MDD, significantly impact sleep quality and daily functioning (30). Nursing interventions addressing sleep-wake cycles may thus represent an important pathway to QoL improvement.

#### 2.2.2 Historical and professional evolution of mental health nursing

The transformation of mental health nursing mirrors the broader evolution in psychiatric care, shifting from institutional models to recovery-oriented, community-based approaches. This paradigm shift has fundamentally redefined the role of nurses, propelling them from passive observers to active participants in therapeutic engagement, while concurrently addressing the systemic fragmentation that characterizes mental healthcare delivery.

##### 2.2.2.1 Transition from custodial care to therapeutic engagement in psychiatric nursing

In the early 20th century, psychiatric nursing was largely centered on custodial care, focusing on patient supervision and the fulfillment of basic needs within the confines of asylum settings. The deinstitutionalization movement of the 1960s–1980s marked a pivotal shift, placing a new emphasis on rehabilitation and community integration (31). This era saw the emergence of nursing practices that integrated biopsychosocial assessments, a stark contrast to the historic symptom-focused checklists (32). Contemporary protocols now routinely incorporate the screening of social determinants of health, addressing aspects of care that were previously overlooked (31).

##### 2.2.2.2 Core competencies defining modern mental health nursing practice

Modern mental health nursing practice is defined by the mastery of six core competencies, as validated by the American Psychiatric Nurses Association (APNA) and WHO guidelines. These competencies are operationalized through frameworks such as the Mental Health Nursing Competency Framework, which links skill acquisition directly to patient outcomes (31).

##### 2.2.2.3 The nurse as a continuity provider in fragmented mental health systems

The fragmentation of mental health services, characterized by isolated service delivery and frequent care transitions, poses a significant barrier to effective management of MDD. Nurses play a crucial role in bridging these gaps through three key mechanisms of continuity: Longitudinal Relationship-Building, Crisis Stabilization, and Care Coordination. Nurses often maintain contact with patients across various settings, including inpatient, outpatient, and community environments. Additionally, nurse-led crisis plans have been shown to shorten emergency response times compared to *ad-hoc* interventions (33).

#### 2.2.3 Classification and application of nursing interventions in depressive disorders

It is important to note that many studies investigating the nursing interventions described below primarily report outcomes such as symptom reduction, treatment adherence, or functional improvement. While these are crucial determinants of QoL, direct evidence from studies using validated, multidimensional QoL instruments as a primary endpoint in MDD-specific populations is more limited. The following sections present these interventions and their observed effects with the understanding that they represent a critical, though incomplete, evidence base for concluding a direct, measured impact on QoL.

The multifaceted nature of depressive disorders necessitates nursing interventions that go beyond pharmacotherapy to address the psychological, social, and functional impairments experienced by patients. Evidence-based nursing strategies are essential for enhancing patient outcomes, targeting the underlying causes of disability in MDD and promoting autonomy and resilience (34, 35).

##### 2.2.3.1 Psychoeducational strategies enhancing patient autonomy and treatment adherence

Psychoeducation is a powerful tool for empowering patients with MDD by providing them with a clear understanding of their condition, its treatment, and self-management techniques. Nurses are instrumental in delivering structured psychoeducational programs that significantly improve medication adherence and reduce the risk of relapse. The evidence supporting these benefits often comes from randomized controlled trials or pre-post studies that primarily use symptom severity and medication adherence rates as their main outcomes (36, 37). However, the direct use of validated QoL scales as a primary endpoint is less common in these studies. Furthermore, limitations such as small sample sizes and insufficient follow-up duration in some trials hinder a robust assessment of the long-term impact of psychoeducation on QoL. Additionally, integrating psychoeducation into routine nursing care has been found to enhance patients’ self-efficacy, particularly when the education is tailored to their cultural and literacy levels (38).

While psychoeducation demonstrates benefits for treatment adherence, the evidence for direct QoL improvement is constrained by methodological limitations. Many studies employ small samples and brief follow-up periods, with QoL measures as secondary outcomes rather than primary endpoints.

##### 2.2.3.2 Nurse-delivered CBT in routine clinical practice

Preliminary evidence from some trials suggests that trained nurses can effectively deliver CBT for depression, with some studies reporting reductions in depressive symptoms that are comparable to those achieved by specialist therapists in specific, often supervised, settings (39, 40). However, the generalizability of these findings is constrained by factors such as variability in nurse training, the level of psychological supervision, and a predominant focus on symptom reduction rather than QoL or functional outcomes.

Common components of nurse-delivered CBT include:

Behavioral activation: Structured scheduling of pleasurable activities to counteract anhedonia.
Cognitive restructuring: Identifying and challenging negative automatic thoughts.
Problem-solving training: Enhancing coping skills for daily stressors.

Despite challenges such as time constraints and limited training, brief, protocol-driven CBT sessions conducted during routine nursing visits have proven to be a feasible approach in reducing symptom severity (38, 40).

The comparative effectiveness of nurse-delivered CBT remains uncertain due to variability in training protocols and supervision. Future trials should prioritize standardized competency frameworks and direct QoL assessment.

##### 2.2.3.3 Integrated care models utilizing nursing-led case management

Nurse-led case management plays a vital role in bridging the gaps within fragmented mental health systems by coordinating multidisciplinary care. For patients with comorbid MDD and chronic illness, nurse case managers have successfully reduced emergency department visits through regular follow-ups and adjustments to care plans (41, 42). The critical functions of nurse case managers include:

Comprehensive assessment: Monitoring both physical and mental health status.

Care coordination: Facilitating communication between psychiatrists, primary care providers, and social workers.

Crisis intervention: Providing immediate support during acute episodes.

Evidence supporting the effectiveness of nurse-led case management on QoL is currently derived mainly from prospective cohort studies or non-randomized trials involving patients with comorbid medical conditions (e.g., cardiovascular disease, cancer) and depressive symptoms (43, 44). For instance, improvements in WHOQOL-Bref scores have been observed in populations like hemodialysis patients with MDD (45, 46). However, these findings must be interpreted with caution. The study designs (often lacking control groups) and the specific nature of the populations (where the physical comorbidity heavily influences QoL) limit the direct applicability of this evidence to primary care populations with standalone MDD. The evidence for case management directly improving QoL in primary MDD populations requires further strengthening through dedicated RCTs.

##### 2.2.3.4 Mind-body interventions administered by nurses to improve daily functioning

Mind-body interventions, including mindfulness-based stress reduction (MBSR) and progressive muscle relaxation, have proven effective in mitigating the somatic symptoms associated with MDD. Nurse-guided MBSR has been found to reduce fatigue and sleep disturbances in participants (47, 48). It is important to note, however, that many supporting studies are pilot feasibility studies that primarily targeted the reduction of anxiety or improvement of sleep as their main outcome. These studies often have small sample sizes and do not exclusively enroll participants with a primary MDD diagnosis. Therefore, the specific effect of these mind-body interventions on the multidimensional QoL of individuals with MDD needs further validation through larger, robust trials that use QoL as a primary endpoint.

Breathing exercises: Taught during bedside care to alleviate acute anxiety.

Guided imagery: Utilized in group settings to enhance emotional regulation.

Yoga therapy: Incorporated into outpatient rehabilitation programs.

In the administration of ketamine, nurses’ monitoring of QTc intervals during infusion ensures patient safety while maximizing the therapeutic benefits of the treatment (49).

##### 2.2.3.5 Family and social reintegration interventions

Beyond individual-focused therapies, nursing interventions that engage the patient’s social environment and functional roles are critical for holistic QoL improvement in MDD. As noted in the conceptualization of QoL, deficits in social relationships and occupational functioning often persist even after symptomatic remission (50). Mental health nurses are uniquely positioned to address these gaps through structured family engagement and community reintegration strategies.

Family Psychoeducation and Support. Involving family members in care is a cornerstone of psychiatric nursing practice. Structured family psychoeducation programs, often nurse-led, aim to improve family understanding of MDD, enhance communication patterns, reduce expressed emotion, and build a more supportive home environment (51). While the primary outcome in many studies has been patient relapse rates or caregiver burden, the improvement in family dynamics and reduction in interpersonal stress are direct contributors to the patient’s perceived social QoL. For instance, programs that equip families with problem-solving skills have been associated with improved patient well-being and family functioning, which are key dimensions of the WHOQOL’s social relationships domain (52).

A summary of the evidence for key nursing interventions discussed in this section, including the target populations, QoL instruments used, nature of findings, and study limitations, is provided in **Table 1**. This table synthesizes the current state of the evidence and highlights the variability in how QoL impacts have been assessed across different intervention types and study designs.

**Table 1:** Summary of evidence on nursing interventions and quality of life (QoL) in Major Depressive Disorder (MDD).

Nursing intervention	Target population	QoL instrument(s) used	Nature and magnitude of oserved QoL change	Main limitations
Psychoeducation	Adults with MDD; Postpartum women	Often inferred from symptom scales (e.g., HAMD) or adherence; Limited use of WHOQOL-Bref	Improvements in QoL domains (e.g., psychological, social) are frequently reported as secondary outcomes or inferred from significant improvements in depressive symptoms and self-efficacy.	Small sample sizes in some trials; Heterogeneous intervention content; Long-term follow-up on QoL is rare; QoL often a secondary outcome.
Nurse-Delivered CBT	Primary care patients with depression; Outpatients with MDD	Primarily symptom measures (BDI, PHQ-9); Direct QoL measures uncommon	Studies primarily demonstrate significant symptom reduction. Improvements in functional domains (a key component of QoL) are reported, but direct, standardized QoL data is limited.	Variability in nurse training and supervision; Focus on symptom reduction over holistic QoL; Generalizability from highly controlled settings.
Integrated Care / Nurse-Led Case Management	Patients with MDD and comorbid physical conditions (e.g., cardiovascular disease, cancer, ESRD)	WHOQOL-Bref [16, 43]; SF-36 [42]	Statistically significant improvements in total QoL scores and specific domains (e.g., psychological, social) observed in cohort studies. Magnitude: Often moderate effect sizes.	Populations with significant physical comorbidities, limiting generalizability to standalone MDD; Lack of RCTs in primary MDD care.
Mind-Body Interventions	Mixed populations (e.g., adults with insomnia, anxiety) often including individuals with depressive symptoms; Not exclusively MDD	PSQI (Sleep Quality); GAD-7 (Anxiety); Direct QoL measures uncommon	Primary outcomes are typically sleeping quality and anxiety reduction. Improvements in these areas are posited to contribute to better overall QoL, but this is not directly measured in most studies.	Small, feasibility-focused studies; Populations not exclusively with primary MDD diagnosis; Lack of MDD-specific QoL outcomes.

#### 2.2.4 Mechanistic pathways linking nursing care to enhanced QoL

The benefits of nursing interventions in MDD extend beyond the alleviation of symptoms to promote fundamental changes in patients’ cognitive processes, behavioral patterns, and social functioning. Three interconnected mechanisms—therapeutic alliance, self-efficacy development, and resilience building—help explain how nurse-led care produces sustained improvements in quality of life.

##### 2.2.4.1 The therapeutic alliance as a catalyst for patient empowerment

The therapeutic alliance between nurse and patient is a foundational component of effective MDD care. It is built on trust, empathy, and shared goal-setting, and distinguishes itself from brief, episodic clinician-patient encounters through the continuity and depth of engagement nurses are able to provide. Observational studies and qualitative research suggest that strong therapeutic alliances are directly associated with higher levels of patient empowerment (39, 53). Within this dynamic, structured psychoeducational interventions illustrate how relationship-building supports recovery. Nurses who provide clear, accessible education about MDD and its underlying mechanisms enable patients to reconceptualize their condition as both understandable and manageable. Programs delivered over a series of structured sessions have been shown to increase patients’ perceived control over their symptoms compared with standard care approaches (54).

##### 2.2.4.2 Development of self-efficacy through sustained nurse-patient interaction

Nurse-delivered CBT, with its emphasis on applied skill-building, is theorized to strengthen self-efficacy. Nurse-delivered CBT, with its emphasis on applied skill-building, actively strengthens self-efficacy more effectively than pharmacotherapy alone. Techniques such as behavioral activation guide patients to reengage in meaningful activities, while systematic cognitive restructuring targets negative thought processes that undermine confidence (55, 56). While these studies primarily demonstrate efficacy for symptom and functional improvement, they provide indirect support for the role of self-efficacy as an underlying mechanism.

Graded task assignments: Breaking larger objectives into achievable steps to maintain momentum and reduce overwhelm.

Mastery experiences: Tracking small but meaningful accomplishments to counteract negative self-perceptions (57).

Verbal persuasion: Offering consistent encouragement, particularly during periods of medication adjustment or challenges with adherence (58).

#### 2.2.5 Barriers to implementation and methodological challenges in current evidence

Despite the demonstrated efficacy of nursing interventions in improving QoL for patients with MDD, several systemic and methodological barriers hinder their widespread adoption. These challenges range from workforce limitations to inconsistencies in outcome measurement, ultimately affecting the scalability and reproducibility of evidence-based practices (59).

##### 2.2.5.1 Systemic constraints including workforce shortages and role ambiguity

Mental health nursing faces chronic workforce shortages, with a scoping review revealing that psychiatric-mental health nurses account for less than the global nursing workforce (60). This deficit is compounded by role ambiguity, where nurses in general medical settings report inadequate training to address complex depressive symptoms, leading to task avoidance or delegation to other specialists (61).

Nurses in psychosocial care networks often perceive mental health interventions as bureaucratic obligations rather than therapeutic opportunities, further reducing implementation fidelity. This systemic undervaluation contrasts with evidence showing nurse-led interventions improve remission rates compared to standard care in primary settings. Role clarification through interdisciplinary care models—such as embedding psychiatric nurses in primary care teams—has shown promise in overcoming these barriers (62).

##### 2.2.5.2 Inconsistencies in QoL measurement tools across clinical studies

The lack of standardized QoL assessment in MDD research obscures comparative effectiveness analyses. While the WHOQOL-Bref is validated for psychiatric populations (17). Methodological advancements are needed to harmonize QoL measurement while preserving patient-centeredness. Initiatives such as the NIH Patient-Reported Outcomes Measurement Information System (PROMIS) offer a modular approach to standardized assessment, allowing for customization while maintaining comparability across studies (62). Future studies should prioritize the use of core outcome sets (COS) to align nursing research with international MDD care standards and facilitate evidence synthesis.

#### 2.2.6 Innovative technologies and nursing interventions

Digital health technologies and artificial intelligence (AI) are revolutionizing the field of mental health care, offering innovative solutions to enhance nursing interventions for patients with MDD (63, 64). These technologies have the potential to improve patient engagement, treatment adherence, and overall quality of life. Additionally, AI algorithms can assist in optimizing resource allocation and tailoring interventions to individual preferences. These advancements in digital health and AI offer promising avenues for improving nursing care and outcomes for patients with MDD (65).

##### 2.2.6.1 Digital health in MDD care

Digital health technologies, including mobile health applications and remote monitoring systems, are increasingly recognized for their potential to enhance nursing interventions in MDD (66). Mobile applications can facilitate symptom tracking, medication reminders, and psychoeducational content delivery, empowering patients to engage actively in self-management between clinical visits (67). These tools also allow nurses to monitor mood fluctuations and treatment adherence in real time, enabling prompt intervention when signs of relapse or deterioration are detected. Remote monitoring platforms can be integrated with wearable devices to track sleep quality, activity levels, and physiological parameters, which provide valuable contextual data for tailoring nursing care plans. Evidence from chronic disease management suggests that digitally supported nursing interventions improve patient engagement and treatment outcomes, which can be translated to mental health care (68).

##### 2.2.6.2 Artificial intelligence for personalized care

AI has the potential to transform nursing interventions in MDD by enabling highly individualized care plans (69). AI-driven predictive models can analyze large datasets — including electronic health records, patient-reported outcomes, and behavioral data from digital health tools — to identify patients at high risk of relapse, non-adherence, or severe symptom exacerbation (70). This predictive capability allows nurses to proactively adjust interventions, such as intensifying psychoeducation, modifying CBT session frequency, or initiating crisis stabilization protocols. AI algorithms can also assist in optimizing resource allocation, matching patients with appropriate community resources, and tailoring interventions to cultural and individual preferences (71). Moreover, natural language processing can be used to analyze patient communication patterns in telehealth or digital platforms to detect subtle mood changes, providing nurses with early warning signals for intervention (72).

#### 2.2.7 Future directions and recommendations

Several evidence gaps and systemic barriers hinder the optimal implementation and evaluation of nursing interventions for QoL improvement in MDD. To address these, a multi-faceted approach is required. We recommend: (1) integrating standardized QoL assessment into nursing education curricula to equip future practitioners with the necessary skills; (2) developing policy frameworks that recognize nurse-led QoL interventions as billable services, thereby ensuring sustainable funding and institutional support; and (3) establishing clear competency pathways for advanced practice mental health nursing to formalize roles in QoL-focused care.

The integration of AI and digital technologies, while promising, presents both opportunities and ethical challenges. Future implementation must carefully consider data privacy, algorithmic bias, and equitable access. Particularly in underserved populations, technology-driven solutions risk exacerbating existing healthcare disparities if not implemented with careful attention to digital literacy and resource availability.

Future research should also prioritize the cultural adaptation and validation of QoL assessment tools, such as the WHOQOL-BREF and PROMIS, for diverse patient populations to ensure their relevance and accuracy. Furthermore, as treatment-resistant depression (TRD) represents a particularly challenging subgroup with severe QoL impairment (73), the development and evaluation of specialized nursing protocols—addressing both novel pharmacological augmentation strategies (74–77)—are urgently needed.

## 3 Conclusion

This review has articulated the pivotal potential and proposed role of nursing interventions in improving the QoL of patients with MDD. However, the evidence base would be strengthened by more studies that employ validated QoL measures as primary outcomes. Moving forward, the integration of innovative technologies offers a promising path ahead. Future research must focus on validating these approaches through robust clinical trials that prioritize standardized QoL measures to firmly establish the holistic impact of nursing care and transform long-term outcomes for individuals living with MDD.

Mechanistic pathways, including the therapeutic alliance, self-efficacy enhancement, and resilience building, provide insight into how nurse-led interventions translate into sustained QoL improvements. However, systemic constraints such as workforce shortages, role ambiguity, variability in intervention fidelity, and inconsistent QoL measurement tools inhibit wider implementation.

The integration of innovative technologies is discussed as a potentially promising path forward. Digital health platforms can extend the reach and continuity of nursing care, while AI can facilitate precision in intervention design and prediction of patient outcomes, supporting timely and personalized care delivery19. Future work must focus on validating these approaches in robust clinical trials while ensuring cultural adaptability. A parallel priority is embedding standardized quality of life (QoL) measures into all intervention protocols. The synthesis of literature presented here suggests that by combining evidence-based therapeutic strategies with technology-driven solutions, nursing practice has the potential to play a more transformative role in achieving holistic recovery and improving long-term outcomes for individuals living with MDD.
